# Crystal structure and Hirshfeld surface analysis of 6-amino-8-phenyl-1,3,4,8-tetra­hydro-2*H*-pyrido[1,2-*a*]pyrimidine-7,9-dicarbo­nitrile

**DOI:** 10.1107/S2056989021003625

**Published:** 2021-04-09

**Authors:** Farid N. Naghiyev, Tatiana A. Tereshina, Victor N. Khrustalev, Mehmet Akkurt, Ali N. Khalilov, Anzurat A. Akobirshoeva, İbrahim G. Mamedov

**Affiliations:** a Department of Chemistry, Baku State University, Z. Khalilov str. 23, Az, 1148 Baku, Azerbaijan; b Peoples’ Friendship University of Russia (RUDN University), Miklukho-Maklay St. 6, Moscow, 117198, Russian Federation; c N. D. Zelinsky Institute of Organic Chemistry RAS, Leninsky Prosp. 47, Moscow, 119991, Russian Federation; d Department of Physics, Faculty of Sciences, Erciyes University, 38039 Kayseri, Turkey; e "Composite Materials" Scientific Research Center, Azerbaijan State Economic University (UNEC), H. Aliyev str. 135, Az 1063, Baku, Azerbaijan; f Acad. Sci. Republ. Tadzhikistan, Kh Yu Yusufbekov Pamir Biol. Inst., 1 Kholdorova St, Khorog 736002, Gbao, Tajikistan

**Keywords:** crystal structure, cyclo­addition product, pyrido[1,2-*a*]pyrimidine, Hirshfeld surface analysis

## Abstract

In the title compound, C_16_H_15_N_5_, the 1,4-di­hydro­pyridine ring has a shallow boat conformation, while the 1,3-diazinane ring adopts an envelope conformation. In the crystal, pairwise N—H⋯N hydrogen bonds generate centrosymmetric dimers featuring *R* ^2^_2_(12) motifs and C—H⋯N contacts connect these dimers to form double layers lying parallel to (001). Weak C—H⋯π and N—H⋯π inter­actions help to consolidate the double layers and van der Waals inter­actions occur between layers. A Hirshfeld surface analysis indicates that the most significant contributions to the crystal packing are from H⋯H (38.5%), N⋯H/H⋯N (33.3%) and C⋯H/H⋯C (27.3%) contacts.

## Abstract 2

In the crystal of the title compound, the mol­ecules form dimers with centrosymmetric 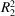
(12) motifs linked by pairwise N—H⋯N hydrogen bonds and C—H⋯N contacts connect these dimers into double layers.

## Chemical context   

Being [6,6]-bicyclic heterocyclic nitro­gen-containing systems, pyrido[1,2-*a*]pyrimidine derivatives are classified as both natural and synthetic compounds and exhibit a broad spectrum of biological properties, such as analgesic, insecticidal, anti-inflammatory, anti­thrombotic, hypoglycaemic and anti­microbial activities (Hermecz & Mészáros, 1988 ▸). The pyrido[1,2-*a*]pyrimidine motif occurs in a number of drugs, such as pemirolast, pirenperone, ramastine, risperidone and paliperidone (Awouters *et al.*, 1986 ▸; Blaton *et al.*, 1995 ▸; Riva *et al.*, 2011 ▸). Two-component and multi-component synthetic methodologies aimed at pyrido[1,2-*a*]pyrimidines as well as their reactions and structural features have been reviewed in the literature (Elattar *et al.*, 2017 ▸).
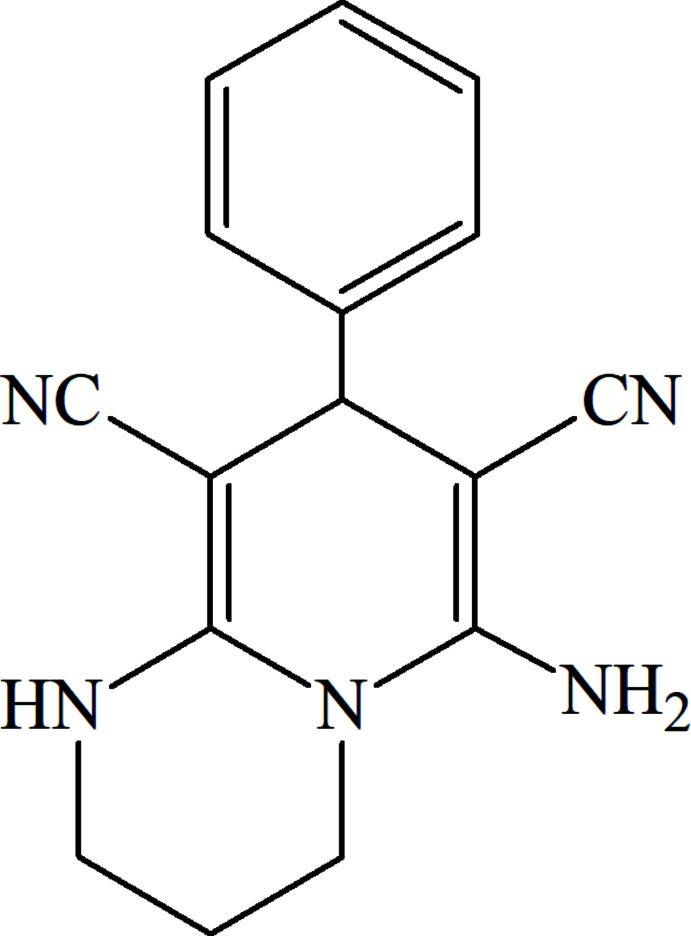


As part of our ongoing studies in this area (Naghiyev *et al.*, 2021 ▸), we now report the crystal structure and Hirshfeld surface analysis of the title compound, C_16_H_15_N_5_ (I), obtained by a three-component synthesis (Naghiyev, 2019 ▸).

## Structural commentary   

**Figure 1:**
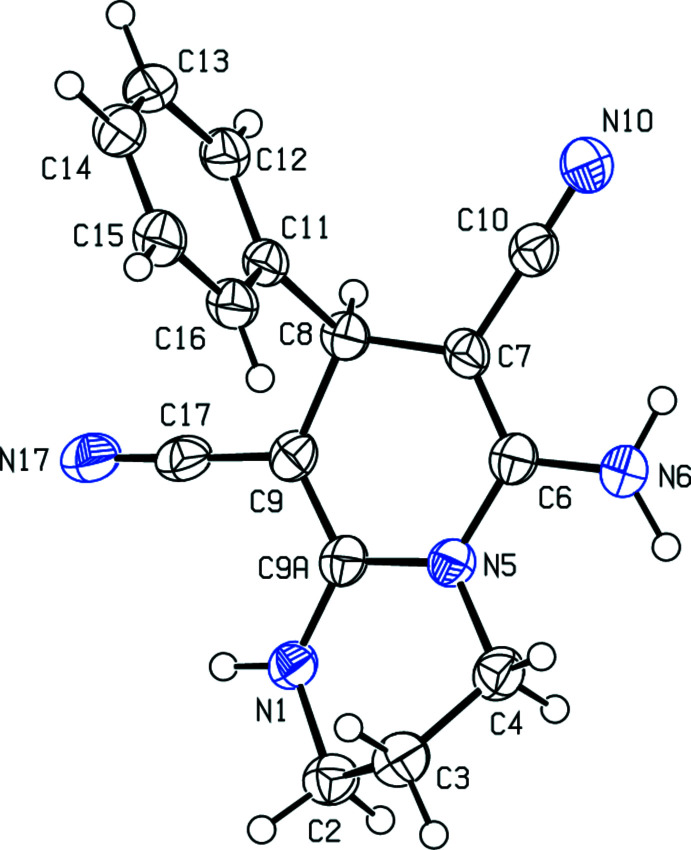
The mol­ecular structure of the title compound with displacement ellipsoids drawn at the 50% probability level.

The 1,4-di­hydro­pyridine ring (N5/C6–C9/C9*A*) of the 1,3,4,8-tetra­hydro-2*H*-pyrido[1,2-*a*]pyrimidine ring system (N1/N5/C2–C4/C6–C9/C9*A*) has a shallow boat conformation with C8 and N5 displaced by 0.094 (3) and 0.075 (2) Å, respectively, from the other four atoms (r.m.s. deviation = 0.011 Å). The 1,3-diazinane ring (N1/N5/C2–C4/C9*A*) adopts an envelope conformation with C3 displaced from the other five atoms (r.m.s. deviation = 0.050 Å) by 0.704 (3) Å. The pendant phenyl ring (C11–C16) subtends a dihedral angle of 89.45 (12)° with the mean plane of the 1,3,4,8-tetra­hydro-2*H*-pyrido[1,2-*a*]pyrimidine ring system (Fig. 1 ▸); C3 and the phenyl ring lie to the same side of the mol­ecule. In the arbitrarily chosen asymmetric mol­ecule, the stereogenic centre C8 has an *R* configuration but crystal symmetry generates a racemic mixture.

## Supra­molecular features   

**Figure 2:**
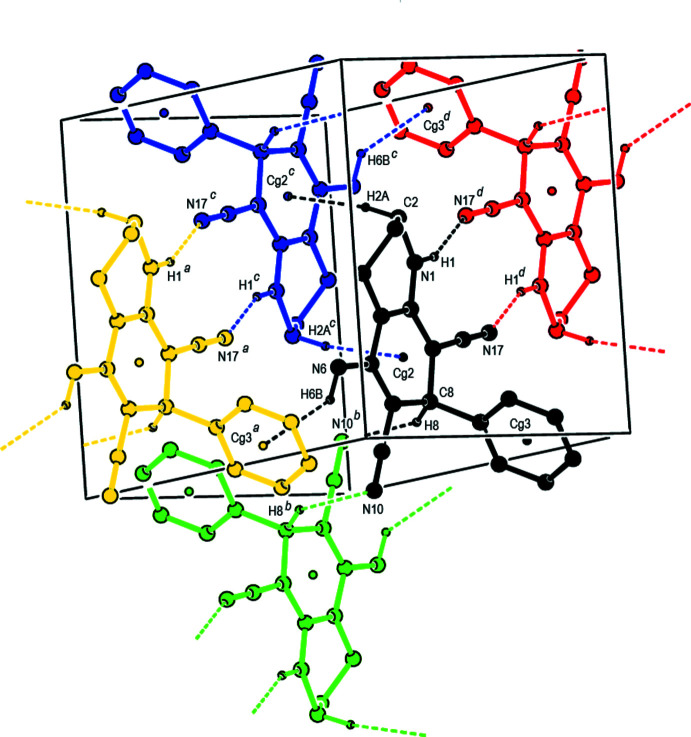
A view of the N—H⋯N, C—H⋯N hydrogen bonds, C—H⋯π and N—H⋯π inter­actions in the extended structure of the title compound. The H atoms not involved in hydrogen bonding have been omitted for clarity. [Symmetry codes: (*a*) −1 + *x*, *y*, *z*; (*b*) 1 − *x*, −*y*, 1 − *z*; (*c*) 1 − *x*, 1 − *y*, 1 − *z*; (*d*) 2 − *x*, 1 − *y*, 1 − *z*].

In the crystal, pairwise N1—H1⋯N17 hydrogen bonds link the mol­ecules into centrosymmetric dimers with 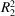
(12) motifs (Table 1 ▸) and C8—H8⋯N10 contacts connect these dimers to form double layers lying parallel to (001) (Figs. 2 ▸ and 3 ▸). The layers are consolidated by C—H⋯π and N—H⋯π inter­actions and weak van der Waals inter­actions occur between the layers.

## Hirshfeld surface analysis   

**Figure 4:**
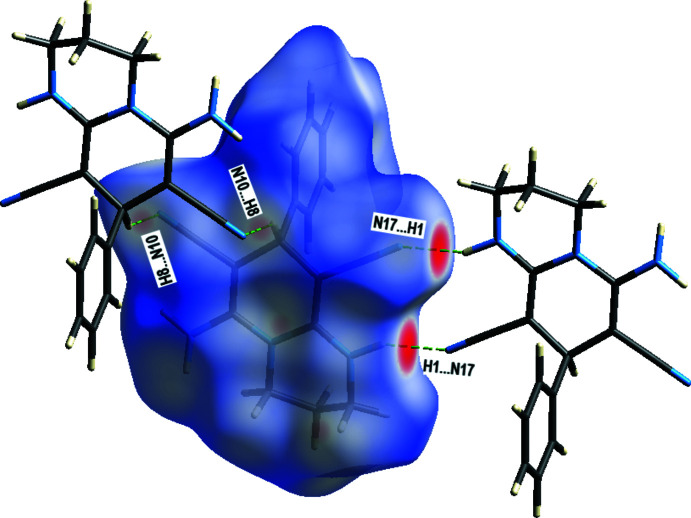
The three-dimensional Hirshfeld surface of the title compound plotted over *d*_norm_ in the range −0.47 to +1.30 a.u.

The nature of the inter­molecular inter­actions in (I) were examined with *CrystalExplorer17.5* (Turner *et al.*, 2017 ▸), using Hirshfeld surfaces (Spackman & Jayatilaka, 2009 ▸) and two-dimensional fingerprint plots. The Hirshfeld surfaces mapped over *d*_norm_ (Fig. 4 ▸) show the inter­molecular contacts as red-coloured spots, which indicate the closer contacts of the N—H⋯N and C—H⋯N hydrogen bonds.

**Figure 5:**
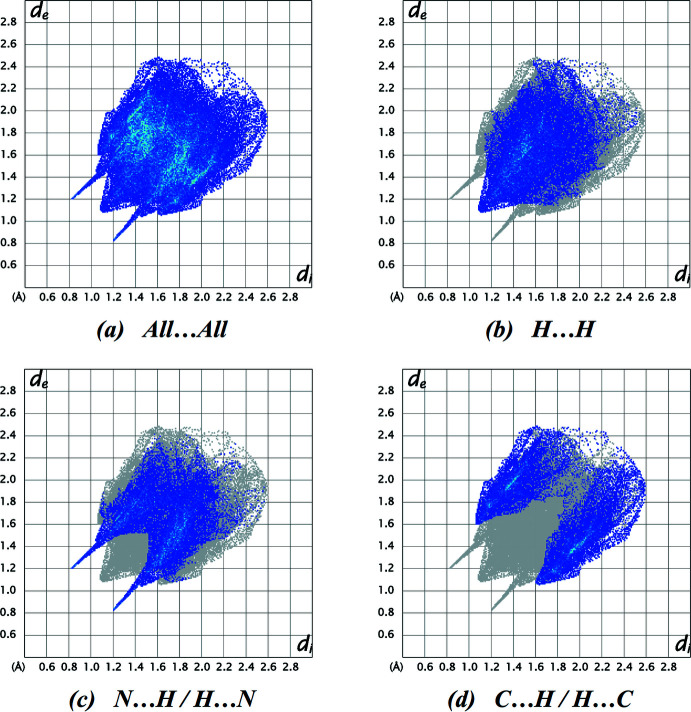
The two-dimensional fingerprint plots of the title compound, showing (*a*) all inter­actions, and delineated into (*b*) H⋯H, (*c*) N⋯H/H⋯N, and (*d*) C⋯H/H⋯C inter­actions.

The two-dimensional fingerprint plots are illustrated in Fig. 5 ▸. H⋯H contacts comprise 38.5% of the total inter­actions, followed by N⋯H/H⋯N (33.3%) and C⋯H/H⋯C (27.3%). The percentage contributions of the N⋯N, C⋯C and C⋯N/N⋯C contacts are negligible, at 0.6, 0.3 and 0.2%, respectively. The predominance of H⋯H, N⋯H/H⋯N and C⋯H/H⋯C contacts indicate that van der Waals inter­actions and hydrogen bonding play the major roles in the crystal packing (Hathwar *et al.*, 2015 ▸).

## Database survey   

The four related compounds containing the 1,3,4,8-tetra­hydro-2*H*-pyrido[1,2-*a*]pyrimidine ring system found in the title compound are 11-(amino­methyl­idene)-8,9,10,11-tetra­hydro­pyrido[2′,3′:4,5]pyrimido[1,2-a]azepin-5(7*H*)-one (Cambridge Structural Database refcode HECLUZ; Khodjaniyazov *et al.*, 2017 ▸), 9-(4-nitro­benzyl­idene)-8,9-di­hydro­pyrido[2,3-*d*]pyrrolo­[1,2-*a*]pyrimidin-5(7*H*)-one (VAMBET; Khodjaniyazov & Ashurov, 2016 ▸), 7′-amino-1′*H*-spiro­[cyclo­heptane-1,2′-pyrim­ido[4,5-*d*]pyrimidin]-4′(3′*H*)-one (LEGLIU; Chen *et al.*, 2012 ▸) and 11-(2-oxopyrrolidin-1-ylmeth­yl)-1,2,3,4,5,6,11,11a-octa­hydro­pyrido[2,1-*b*]quinazolin-6-one dihydrate (KUTPEV; Samarov *et al.*, 2010 ▸).

In the mol­ecule of HECLUZ, the seven-membered penta­methyl­ene ring adopts a twist-boat conformation. In the crystal, hydrogen bonds with a 16-membered ring and a chain motif are generated by N—H⋯N and N—H⋯O contacts. The hydrogen-bonded chains formed along [100] are connected by aromatic π–π stacking inter­actions observed between the pyridine and pyrimidine rings. In the crystal of VAMBET, the mol­ecules are linked *via* C—H⋯O and C—H⋯N hydrogen bonds, forming layers lying parallel to (101). In LEGLIU, the mol­ecular structure is built up with two fused six-membered rings and one seven-membered ring linked through a spiro C atom. The crystal packing features N—H⋯O hydrogen bonds. In KUTPEV, the water mol­ecules are mutually O—H⋯O hydrogen bonded and form infinite chains propagating along the *b-*axis direction. Neighboring chains are linked by the quinazoline mol­ecules by means of O—H⋯O=C hydrogen bonds, forming a two-dimensional network.

## Synthesis and crystallization   

The title compound was synthesized using our previously reported procedure (Naghiyev, 2019 ▸), and colourless prisms were obtained upon recrystallization from methanol solution.

## Refinement   

Crystal data, data collection and structure refinement details are summarized in Table 2 ▸. The C-bound H atoms were placed in calculated positions (C—H = 0.95–1.00 Å) and refined as riding atoms with *U*_iso_(H) = 1.2*U*_eq_(C). The N-bound H atoms were located in difference maps and their positions were freely refined with the constraint *U*_iso_(H) = 1.2*U*_eq_(N) applied.

## Supplementary Material

Crystal structure: contains datablock(s) I. DOI: 10.1107/S2056989021003625/hb7972sup1.cif

Structure factors: contains datablock(s) I. DOI: 10.1107/S2056989021003625/hb7972Isup2.hkl

CCDC reference: 2075718

Additional supporting information:  crystallographic information; 3D view; checkCIF report

## Figures and Tables

**Figure 3:**
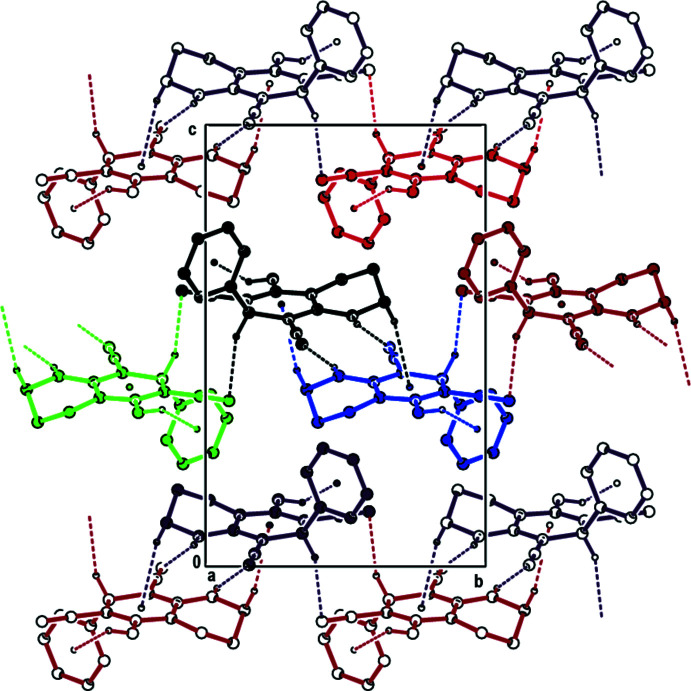
View down [100] showing the formation of (001) layers in the title compound by means of N—H⋯N, C—H⋯N, C—H⋯π and N—H⋯π inter­actions.

**Table 1:** Hydrogen-bond geometry (Å, °) *Cg*2 and *Cg*3 are the centroids of the N5/C6–C9/C9*A* pyridine ring and the C11–C16 phenyl ring, respectively.

*D*—H⋯*A*	*D*—H	H⋯*A*	*D*⋯*A*	*D*—H⋯*A*
N1—H1⋯N17^i^	0.87 (3)	2.15 (3)	2.975 (4)	157 (3)
C8—H8⋯N10^ii^	1.00	2.57	3.447 (4)	146
C2—H2*A*⋯*Cg*2^iii^	0.99	2.67	3.620 (3)	161
N6—H6*B*⋯*Cg*3^iv^	0.92 (4)	2.98 (3)	3.633 (3)	129 (3)

Symmetry codes: (i) -x+2, -y+1, -z+1; (ii) -x+1, -y, -z+1; (iii) -x+1, -y+1, -z+1; (iv) x-1, y, z.

**Table 2:** Experimental details

Crystal data	
Chemical formula	C_16_H_15_N_5_
*M*_r_	277.33
Crystal system, space group	Monoclinic, *P*2_1_/*c*
Temperature (K)	100
*a*, *b*, *c* (Å)	8.2521 (6), 10.2774 (8), 16.2102 (12)
β (°)	92.070 (2)
*V* (Å^3^)	1373.89 (18)
*Z*	4
Radiation type	Mo *K*α
μ (mm^−1^)	0.09
Crystal size (mm)	0.12 × 0.06 × 0.04

Data collection	
Diffractometer	Bruker D8 QUEST PHOTON-III CCD
Absorption correction	Multi-scan (*SADABS*; Krause *et al.*, 2015 ▸)
*T*_min_, *T*_max_	0.981, 0.990
No. of measured, independent and observed [*I* > 2σ(*I*)] reflections	21387, 3146, 1519
*R*_int_	0.104
(sin θ/λ)_max_ (Å^−1^)	0.649

Refinement	
*R*[*F* ^2^ > 2σ(*F* ^2^)], *wR*(*F* ^2^), *S*	0.066, 0.172, 1.01
No. of reflections	3146
No. of parameters	200
H-atom treatment	H atoms treated by a mixture of independent and constrained refinement
Δρ_max_, Δρ_min_ (e Å^−3^)	0.30, −0.27

Computer programs: *APEX3* (Bruker, 2018 ▸), *SAINT* (Bruker, 2013 ▸), *SHELXT2014/5* (Sheldrick, 2015*a* ▸), *SHELXL2018/3* (Sheldrick, 2015*b* ▸), *ORTEP-3 for Windows* (Farrugia, 2012 ▸) and *PLATON* (Spek, 2020 ▸).

## supplementary crystallographic information

### Crystal data

**Table:**

C_16_H_15_N_5_	*F*(000) = 584
*M**_r_* = 277.33	*D*_x_ = 1.341 Mg m^−^^3^
Monoclinic, *P*2_1_/*c*	Mo *K*α radiation, λ = 0.71073 Å
*a* = 8.2521 (6) Å	Cell parameters from 1452 reflections
*b* = 10.2774 (8) Å	θ = 2.4–22.3°
*c* = 16.2102 (12) Å	µ = 0.09 mm^−^^1^
β = 92.070 (2)°	*T* = 100 K
*V* = 1373.89 (18) Å^3^	Prism, colourless
*Z* = 4	0.12 × 0.06 × 0.04 mm

### Data collection

**Table:**

Bruker D8 QUEST PHOTON-III CCD diffractometer	1519 reflections with *I* > 2σ(*I*)
φ and ω scans	*R*_int_ = 0.104
Absorption correction: multi-scan (SADABS; Krause *et al.*, 2015)	θ_max_ = 27.5°, θ_min_ = 2.4°
*T*_min_ = 0.981, *T*_max_ = 0.990	*h* = −10→10
21387 measured reflections	*k* = −13→13
3146 independent reflections	*l* = −21→21

### Refinement

**Table:**

Refinement on *F*^2^	Secondary atom site location: difference Fourier map
Least-squares matrix: full	Hydrogen site location: mixed
*R*[*F*^2^ > 2σ(*F*^2^)] = 0.066	H atoms treated by a mixture of independent and constrained refinement
*wR*(*F*^2^) = 0.172	*w* = 1/[σ^2^(*F*_o_^2^) + (0.0647*P*)^2^ + 0.4109*P*] where *P* = (*F*_o_^2^ + 2*F*_c_^2^)/3
*S* = 1.01	(Δ/σ)_max_ < 0.001
3146 reflections	Δρ_max_ = 0.30 e Å^−^^3^
200 parameters	Δρ_min_ = −0.27 e Å^−^^3^
0 restraints	Extinction correction: SHELXL, Fc^*^=kFc[1+0.001xFc^2^λ^3^/sin(2θ)]^-1/4^
Primary atom site location: difference Fourier map	Extinction coefficient: 0.00309 (14)

### Special details

**Table:**

Geometry. All esds (except the esd in the dihedral angle between two l.s. planes) are estimated using the full covariance matrix. The cell esds are taken into account individually in the estimation of esds in distances, angles and torsion angles; correlations between esds in cell parameters are only used when they are defined by crystal symmetry. An approximate (isotropic) treatment of cell esds is used for estimating esds involving l.s. planes.

### Fractional atomic coordinates and isotropic or equivalent isotropic displacement parameters (Å^2^)

**Table:**

	*x*	*y*	*z*	*U*_iso_*/*U*_eq_	
N1	0.6921 (3)	0.5239 (3)	0.56852 (16)	0.0325 (7)	
H1	0.787 (4)	0.541 (3)	0.549 (2)	0.039*	
C2	0.6082 (4)	0.6387 (3)	0.5990 (2)	0.0376 (8)	
H2A	0.5337	0.6740	0.5553	0.045*	
H2B	0.6879	0.7071	0.6148	0.045*	
C3	0.5135 (4)	0.5990 (3)	0.6733 (2)	0.0401 (9)	
H3A	0.5893	0.5737	0.7193	0.048*	
H3B	0.4474	0.6731	0.6918	0.048*	
C4	0.4049 (4)	0.4861 (3)	0.6502 (2)	0.0376 (8)	
H4A	0.3478	0.4564	0.6995	0.045*	
H4B	0.3224	0.5144	0.6082	0.045*	
N5	0.5002 (3)	0.3771 (2)	0.61711 (15)	0.0317 (6)	
C6	0.4428 (4)	0.2509 (3)	0.62264 (19)	0.0321 (8)	
N6	0.2881 (3)	0.2398 (3)	0.65141 (19)	0.0388 (7)	
H6A	0.211 (4)	0.313 (4)	0.650 (2)	0.047*	
H6B	0.255 (4)	0.155 (4)	0.645 (2)	0.047*	
C7	0.5356 (4)	0.1470 (3)	0.60400 (19)	0.0306 (7)	
C8	0.7070 (4)	0.1551 (3)	0.57515 (18)	0.0298 (7)	
H8	0.7106	0.1095	0.5208	0.036*	
C9	0.7468 (4)	0.2969 (3)	0.56112 (19)	0.0315 (8)	
C9A	0.6496 (4)	0.3993 (3)	0.58218 (18)	0.0309 (7)	
C10	0.4710 (4)	0.0202 (3)	0.6141 (2)	0.0340 (8)	
N10	0.4207 (3)	−0.0837 (3)	0.62453 (19)	0.0443 (8)	
C11	0.8298 (4)	0.0898 (3)	0.63415 (19)	0.0297 (7)	
C12	0.9153 (4)	−0.0185 (3)	0.6105 (2)	0.0363 (8)	
H12	0.8957	−0.0541	0.5570	0.044*	
C13	1.0300 (4)	−0.0762 (3)	0.6639 (2)	0.0421 (9)	
H13	1.0905	−0.1492	0.6465	0.051*	
C14	1.0550 (4)	−0.0269 (3)	0.7422 (2)	0.0431 (9)	
H14	1.1318	−0.0671	0.7791	0.052*	
C15	0.9700 (4)	0.0802 (3)	0.7676 (2)	0.0402 (9)	
H15	0.9878	0.1139	0.8218	0.048*	
C16	0.8582 (4)	0.1383 (3)	0.7135 (2)	0.0355 (8)	
H16	0.7998	0.2125	0.7309	0.043*	
C17	0.8973 (4)	0.3241 (3)	0.5286 (2)	0.0360 (8)	
N17	1.0252 (4)	0.3441 (3)	0.5036 (2)	0.0497 (8)	

### Atomic displacement parameters (Å^2^)

**Table:**

	*U*^11^	*U*^22^	*U*^33^	*U*^12^	*U*^13^	*U*^23^
N1	0.0306 (16)	0.0316 (16)	0.0353 (16)	0.0031 (12)	0.0009 (12)	0.0023 (12)
C2	0.041 (2)	0.0356 (19)	0.0359 (19)	0.0034 (16)	−0.0041 (16)	−0.0013 (16)
C3	0.042 (2)	0.042 (2)	0.0356 (19)	0.0054 (17)	−0.0035 (16)	−0.0043 (16)
C4	0.0335 (19)	0.040 (2)	0.0393 (19)	0.0035 (15)	−0.0024 (15)	−0.0017 (16)
N5	0.0302 (15)	0.0310 (16)	0.0334 (15)	0.0014 (12)	−0.0038 (12)	0.0009 (12)
C6	0.0310 (18)	0.0353 (19)	0.0294 (17)	0.0014 (15)	−0.0098 (14)	0.0014 (14)
N6	0.0293 (17)	0.0360 (17)	0.0509 (19)	0.0008 (13)	−0.0005 (13)	0.0006 (15)
C7	0.0296 (18)	0.0308 (18)	0.0306 (17)	−0.0042 (14)	−0.0096 (14)	0.0026 (14)
C8	0.0323 (18)	0.0305 (18)	0.0260 (17)	−0.0006 (14)	−0.0079 (13)	0.0003 (13)
C9	0.0297 (18)	0.0352 (19)	0.0290 (17)	0.0027 (14)	−0.0068 (14)	0.0039 (14)
C9A	0.0302 (18)	0.035 (2)	0.0269 (17)	0.0024 (15)	−0.0100 (14)	0.0016 (14)
C10	0.0317 (19)	0.038 (2)	0.0314 (18)	0.0015 (16)	−0.0062 (14)	−0.0029 (15)
N10	0.0382 (17)	0.0407 (18)	0.053 (2)	−0.0013 (15)	−0.0098 (14)	−0.0078 (15)
C11	0.0287 (17)	0.0282 (17)	0.0316 (17)	−0.0019 (14)	−0.0062 (13)	0.0024 (14)
C12	0.0342 (19)	0.0344 (19)	0.040 (2)	−0.0027 (15)	−0.0056 (15)	−0.0005 (15)
C13	0.038 (2)	0.0316 (19)	0.056 (2)	0.0041 (15)	−0.0080 (17)	0.0046 (17)
C14	0.038 (2)	0.036 (2)	0.054 (2)	0.0006 (16)	−0.0195 (18)	0.0087 (17)
C15	0.040 (2)	0.041 (2)	0.039 (2)	−0.0029 (17)	−0.0148 (16)	0.0007 (16)
C16	0.0371 (19)	0.0320 (18)	0.0366 (19)	−0.0019 (15)	−0.0114 (15)	−0.0004 (15)
C17	0.040 (2)	0.0296 (19)	0.038 (2)	0.0077 (15)	−0.0040 (16)	0.0038 (15)
N17	0.0442 (19)	0.0336 (18)	0.072 (2)	0.0075 (14)	0.0096 (17)	0.0112 (16)

### Geometric parameters (Å, º)

**Table:**

N1—C9A	1.349 (4)	C7—C8	1.508 (4)
N1—C2	1.463 (4)	C8—C9	1.513 (4)
N1—H1	0.87 (3)	C8—C11	1.524 (4)
C2—C3	1.515 (5)	C8—H8	1.0000
C2—H2A	0.9900	C9—C9A	1.373 (4)
C2—H2B	0.9900	C9—C17	1.395 (5)
C3—C4	1.505 (4)	C10—N10	1.160 (4)
C3—H3A	0.9900	C11—C12	1.379 (4)
C3—H3B	0.9900	C11—C16	1.392 (4)
C4—N5	1.481 (4)	C12—C13	1.393 (4)
C4—H4A	0.9900	C12—H12	0.9500
C4—H4B	0.9900	C13—C14	1.375 (5)
N5—C6	1.384 (4)	C13—H13	0.9500
N5—C9A	1.394 (4)	C14—C15	1.376 (5)
C6—C7	1.355 (4)	C14—H14	0.9500
C6—N6	1.379 (4)	C15—C16	1.385 (4)
N6—H6A	0.99 (4)	C15—H15	0.9500
N6—H6B	0.92 (4)	C16—H16	0.9500
C7—C10	1.420 (5)	C17—N17	1.162 (4)
			
C9A—N1—C2	125.6 (3)	C7—C8—C9	108.2 (3)
C9A—N1—H1	119 (2)	C7—C8—C11	113.0 (2)
C2—N1—H1	114 (2)	C9—C8—C11	112.1 (2)
N1—C2—C3	108.4 (3)	C7—C8—H8	107.8
N1—C2—H2A	110.0	C9—C8—H8	107.8
C3—C2—H2A	110.0	C11—C8—H8	107.8
N1—C2—H2B	110.0	C9A—C9—C17	118.5 (3)
C3—C2—H2B	110.0	C9A—C9—C8	124.6 (3)
H2A—C2—H2B	108.4	C17—C9—C8	116.8 (3)
C4—C3—C2	109.2 (3)	N1—C9A—C9	122.0 (3)
C4—C3—H3A	109.8	N1—C9A—N5	117.4 (3)
C2—C3—H3A	109.8	C9—C9A—N5	120.6 (3)
C4—C3—H3B	109.8	N10—C10—C7	178.0 (3)
C2—C3—H3B	109.8	C12—C11—C16	118.4 (3)
H3A—C3—H3B	108.3	C12—C11—C8	121.1 (3)
N5—C4—C3	110.8 (3)	C16—C11—C8	120.5 (3)
N5—C4—H4A	109.5	C11—C12—C13	120.9 (3)
C3—C4—H4A	109.5	C11—C12—H12	119.6
N5—C4—H4B	109.5	C13—C12—H12	119.6
C3—C4—H4B	109.5	C14—C13—C12	119.6 (3)
H4A—C4—H4B	108.1	C14—C13—H13	120.2
C6—N5—C9A	119.3 (3)	C12—C13—H13	120.2
C6—N5—C4	119.9 (3)	C13—C14—C15	120.7 (3)
C9A—N5—C4	120.8 (3)	C13—C14—H14	119.7
C7—C6—N6	123.2 (3)	C15—C14—H14	119.7
C7—C6—N5	121.8 (3)	C14—C15—C16	119.4 (3)
N6—C6—N5	115.0 (3)	C14—C15—H15	120.3
C6—N6—H6A	122 (2)	C16—C15—H15	120.3
C6—N6—H6B	109 (2)	C15—C16—C11	121.1 (3)
H6A—N6—H6B	122 (3)	C15—C16—H16	119.4
C6—C7—C10	118.7 (3)	C11—C16—H16	119.4
C6—C7—C8	124.7 (3)	N17—C17—C9	177.7 (4)
C10—C7—C8	116.6 (3)		
			
C9A—N1—C2—C3	22.3 (4)	C2—N1—C9A—N5	10.5 (4)
N1—C2—C3—C4	−54.1 (3)	C17—C9—C9A—N1	3.4 (5)
C2—C3—C4—N5	55.8 (4)	C8—C9—C9A—N1	179.6 (3)
C3—C4—N5—C6	154.4 (3)	C17—C9—C9A—N5	−178.1 (3)
C3—C4—N5—C9A	−23.9 (4)	C8—C9—C9A—N5	−1.9 (5)
C9A—N5—C6—C7	7.8 (4)	C6—N5—C9A—N1	172.0 (3)
C4—N5—C6—C7	−170.5 (3)	C4—N5—C9A—N1	−9.7 (4)
C9A—N5—C6—N6	−174.6 (3)	C6—N5—C9A—C9	−6.6 (4)
C4—N5—C6—N6	7.1 (4)	C4—N5—C9A—C9	171.7 (3)
N6—C6—C7—C10	0.3 (5)	C7—C8—C11—C12	115.1 (3)
N5—C6—C7—C10	177.6 (3)	C9—C8—C11—C12	−122.4 (3)
N6—C6—C7—C8	−177.9 (3)	C7—C8—C11—C16	−64.8 (4)
N5—C6—C7—C8	−0.5 (5)	C9—C8—C11—C16	57.7 (4)
C6—C7—C8—C9	−6.8 (4)	C16—C11—C12—C13	−1.4 (5)
C10—C7—C8—C9	175.0 (3)	C8—C11—C12—C13	178.6 (3)
C6—C7—C8—C11	117.8 (3)	C11—C12—C13—C14	1.8 (5)
C10—C7—C8—C11	−60.3 (4)	C12—C13—C14—C15	−1.1 (5)
C7—C8—C9—C9A	8.0 (4)	C13—C14—C15—C16	0.0 (5)
C11—C8—C9—C9A	−117.3 (3)	C14—C15—C16—C11	0.4 (5)
C7—C8—C9—C17	−175.7 (3)	C12—C11—C16—C15	0.3 (5)
C11—C8—C9—C17	59.1 (4)	C8—C11—C16—C15	−179.7 (3)
C2—N1—C9A—C9	−170.9 (3)		

### Hydrogen-bond geometry (Å, º)

*Cg*2 and *Cg*3 are the centroids of the N5/C6–C9/C9*A* pyridine ring and the C11–C16 phenyl ring, respectively.

**Table:**

*D*—H···*A*	*D*—H	H···*A*	*D*···*A*	*D*—H···*A*
N1—H1···N17^i^	0.87 (3)	2.15 (3)	2.975 (4)	157 (3)
C8—H8···N10^ii^	1.00	2.57	3.447 (4)	146
C2—H2*A*···*Cg*2^iii^	0.99	2.67	3.620 (3)	161
N6—H6*B*···*Cg*3^iv^	0.92 (4)	2.98 (3)	3.633 (3)	129 (3)

Symmetry codes: (i) −*x*+2, −*y*+1, −*z*+1; (ii) −*x*+1, −*y*, −*z*+1; (iii) −*x*+1, −*y*+1, −*z*+1; (iv) *x*−1, *y*, *z*.

## References

[bb1] Awouters, F., Vermeire, J., Smeyers, F., Vermote, P., van Beek, R. & Niemegeers, C. J. E. (1986). *Drug Dev. Res.* **8**, 95–102.

[bb2] Blaton, N. M., Peeters, O. M. & De Ranter, C. J. (1995). *Acta Cryst.* C**51**, 533–535.

[bb20] Bruker (2018). *APEX3*. Bruker AXS Inc., Madison, Wisconsin, USA.

[bb21] Bruker (2013). *SAINT*. Bruker AXS Inc., Madison, Wisconsin, USA.

[bb3] Chen, S., Shi, D., Liu, M. & Li, J. (2012). *Acta Cryst.* E**68**, o2546. 10.1107/S1600536812031492 PMC3414991 22904978

[bb4] Elattar, K. M., Rabie, R. & Hammouda, M. M. (2017). *Monatsh. Chem.* **148**, 601–627.

[bb5] Farrugia, L. J. (2012). *J. Appl. Cryst.* **45**, 849–854.

[bb6] Hathwar, V. R., Sist, M., Jørgensen, M. R. V., Mamakhel, A. H., Wang, X., Hoffmann, C. M., Sugimoto, K., Overgaard, J. & Iversen, B. B. (2015). *IUCrJ*, **2**, 563–574. 10.1107/S2052252515012130 PMC4547824 26306198

[bb7] Hermecz, I. & Mészáros, Z. (1988). *Med. Res. Rev.* **8**, 203–230. 10.1002/med.2610080204 3288821

[bb8] Khodjaniyazov, Kh. U. & Ashurov, J. M. (2016). *Acta Cryst.* E**72**, 452–455. 10.1107/S2056989016003583 PMC4910333 27375862

[bb9] Khodjaniyazov, K. U., Makhmudov, U. S., Turgunov, K. K. & Elmuradov, B. Z. (2017). *Acta Cryst.* E**73**, 1497–1500. 10.1107/S2056989017013093 PMC5730303 29250366

[bb10] Krause, L., Herbst-Irmer, R., Sheldrick, G. M. & Stalke, D. (2015). *J. Appl. Cryst.* **48**, 3–10. 10.1107/S1600576714022985 PMC4453166 26089746

[bb11] Naghiyev, F. N. (2019). *Chem. Probl.* **17**, 275–281.

[bb12] Naghiyev, F. N., Grishina, M. M., Khrustalev, V. N., Khalilov, A. N., Akkurt, M., Akobirshoeva, A. A. & Mamedov, İ. G. (2021). *Acta Cryst.* E**77**, 195–199. 10.1107/S2056989021000785 PMC7869549 33614153

[bb13] Riva, R., Banfi, L., Castaldi, G., Ghislieri, D., Malpezzi, L., Musumeci, F., Tufaro, R. & Rasparini, M. (2011). *Eur. J. Org. Chem.* pp. 2319–2325.

[bb14] Samarov, Z. U., Okmanov, R. Y., Turgunov, K. K., Tashkhodjaev, B. & Shakhidoyatov, K. M. (2010). *Acta Cryst.* E**66**, o890. 10.1107/S1600536810009955 PMC2983797 21580707

[bb15] Sheldrick, G. M. (2015*a*). *Acta Cryst.* A**71**, 3–8.

[bb16] Sheldrick, G. M. (2015*b*). *Acta Cryst.* C**71**, 3–8.

[bb17] Spackman, M. A. & Jayatilaka, D. (2009). *CrystEngComm*, **11**, 19–32.

[bb18] Spek, A. L. (2020). *Acta Cryst.* E**76**, 1–11. 10.1107/S2056989019016244 PMC6944088 31921444

[bb19] Turner, M. J., Mckinnon, J. J., Wolff, S. K., Grimwood, D. J., Spackman, P. R., Jayatilaka, D. & Spackman, M. A. (2017). *CrystalExplorer*17. University of Western Australia.

